# Schwartz Rounds for Staff in an Australian Tertiary Hospital: Protocol for a Pilot Uncontrolled Trial

**DOI:** 10.2196/35083

**Published:** 2022-04-27

**Authors:** Tatjana Ewais, Georgia Hunt, Jonathan Munro, Paul Pun, Christy Hogan, Leeroy William, Andrew Teodorczuk

**Affiliations:** 1 Mater Young Adult Health Centre Mater Health Mater Misericordiae Ltd South Brisbane Australia; 2 School of Medicine and Dentistry Griffith University Gold Coast Australia; 3 Mater Clinical School Faculty of Medicine University of Queensland South Brisbane Australia; 4 Mater in Mind Mater Health Mater Misericordiae Ltd South Brisbane Australia; 5 Eastern Health Clinical School Faculty of Medicine, Nursing and Health Sciences Monash University Melbourne Australia

**Keywords:** Schwartz Rounds, compassionate care, health care staff well-being

## Abstract

**Background:**

Schwartz Rounds are a unique, organization-wide interdisciplinary intervention aimed at enhancing staff well-being, compassionate care, teamwork, and organizational culture in health care settings. They provide a safe space wherein both clinical and nonclinical health staff can connect and share their experiences about the social and emotional aspects of health care.

**Objective:**

Although Schwartz Rounds have been assessed and widely implemented in the United States and United Kingdom, they are yet to be formally evaluated in Australian health care settings. The purpose of this study is to evaluate the feasibility and impact of Schwartz Rounds on staff well-being, compassionate care, and organizational culture, in a tertiary metropolitan hospital in Brisbane, Australia.

**Methods:**

This mixed methods repeated measures pilot study will recruit 24 participants in 2 groups from 2 departments, the intensive care unit and the gastroenterology department. Participants from each group will take part in 3 unit-based Schwartz Rounds. Primary outcomes will include the study and intervention feasibility measures, while secondary outcomes will include scores on the Maslach Burnout Inventory–Human Services Survey, the Schwartz Centre Compassionate Care Scale, and the Culture of Care Barometer. Primary and secondary outcomes will be collected at baseline, after the Rounds, and 3-month follow-up. Two focus groups will be held approximately 2 months after completion of the Schwartz Rounds. Descriptive statistics, paired t tests, chi-square tests, and analysis of variance will be used to compare quantitative data across time points and groups. Qualitative data from focus groups and free-text survey questions will be analyzed using an inductive thematic analysis approach.

**Results:**

The study was approved by the Mater Hospital Human Research Ethics Committee (reference number: HREC/MML/71868) and recruitment commenced in July 2021; study completion is anticipated by May 2022.

**Conclusions:**

The study will contribute to the assessment of feasibility and preliminary efficacy of the Schwartz Rounds in a tertiary Australian hospital during the COVID-19 pandemic.

**Trial Registration:**

Australian New Zealand Clinical Trials Registry ACTRN12621001473853; https://www.anzctr.org.au/Trial/Registration/TrialReview.aspx?id=382769&isReview=true

**International Registered Report Identifier (IRRID):**

DERR1-10.2196/35083

## Introduction

### Background

Employees working within health care settings are at greater risk of mental health concerns compared with the general public. Health care workers have been found to experience high rates of work-related stress, burnout, depression, anxiety, and suicidal ideation [1-3]. This has also been shown to impact the quality of patient care and compassionate care [4]. Furthermore, the current COVID-19 pandemic has brought into focus the well-being of health care staff and compassionate care with unprecedented challenges across health care settings, increased workload, uncertainty, and stress [5]. It has highlighted the need for interventions aimed at preventing and treating psychological distress and disorders in health care workers and improving organizational support and culture [5].

Recently, an Australian framework, named “Every Doctor, Every Setting,” has been designed to support the mental health and well-being of doctors and medical students [6]. Its guiding principle is that improving the well-being of doctors and medical students is a key enabler of good patient care. The framework outlines that, in addition to strategies to increase the well-being of health professionals, strategies that focus on improving team and system cultures are to be promoted, as individual-level approaches often fail to be translated into practice benefits due to organizational cultural barriers. This is supported by the literature on the importance of health care worker support, within an environment that is conducive to open discussions about the social and emotional aspects of their work [7].

Various interventions have been implemented within health care settings to increase empathy and improve patient care, reduce work stress and burnout, and improve staff well-being [6-8]. While many interventions have been evaluated, few allow for organization-wide involvement (ie, multiple disciplines, both clinical and nonclinical staff), most might be one-off events, that rely solely on individual involvement (ie, counseling), and require all attendees to participate. Therefore, there is a pressing need for interventions in health care that are focused on the social and emotional aspects of care, that are ongoing, allow all staff to attend and choose to participate, and require engagement by both individual staff and the larger institution. One such format that provides a safe space for both clinical and nonclinical health care staff is Schwartz Rounds [9,10].

### Schwartz Rounds in Health Care Settings

Schwartz Rounds have been developed by the Schwartz Centre for Compassionate Care as a unique intervention where health care workers can share and reflect on their experiences of the social and emotional aspects of health care [10]. Schwartz Rounds (hereafter the Rounds) were inspired by the late Kenneth Schwartz who recognized that his own cancer care was improved through authentic, individualized, and compassionate care by health care workers from all professions.

To facilitate Hospital staff attendance and participation, the Rounds were designed to follow the Hospital medical rounds structure in that they have a topic, a panel, and an audience; they are conducted monthly (for approximately 1 hour), and usually during the lunch time and with food provided for the participants. The key distinguishing features of the Rounds are that their content is focused on the emotional and social aspects of care rather than the clinical aspects, and that they are open to all staff, clinical and nonclinical. The purpose of the Rounds as an intervention aimed at exploring and sharing the emotional and social challenges of providing care, rather than solving problems or debriefing, is stated at the outset of every Round. The Rounds start with a brief introduction of their history and purpose. This is followed by 2-4 panelists sharing their stories about the emotional aspects of care for about 5 minutes each, and a facilitated, reflective discussion where the participants share their thoughts and reflections on the content heard.

There is evidence that the Rounds have a positive impact within hospital and educational settings for staff, patients, and organizations [11,12]. They have been reported to increase compassionate patient care; improve teamwork and staff relations; improve organizational and institutional culture (ie, enhanced patient-centered care and shared purpose); normalize and validate emotional reactions and eradicate the stigma of emotional responses within a health care setting; and positively impact staff through reducing their work-related stress, and improved psychological well-being and ability to respond to challenges [13-25]. The Rounds have been shown to be sustainable with organizational support, strong leadership, and a committee designed to run and organize the Rounds. They have been widely implemented in the Unites States and United Kingdom and are starting to be implemented in other countries including Canada, Ireland, New Zealand, and Australia.

Despite the considerable evidence regarding the benefits of the Rounds, a recent systematic review has identified that the overall level of evidence is low to moderate with many of the studies lacking methodological rigor [8]. Furthermore, the review did not find any research examining the feasibility and efficacy of Schwartz Rounds in Australian health care settings. This study protocol is based on the known process and features of the Rounds but it was developed to suit an Australian metropolitan tertiary hospital. The protocol incorporates a COVID-19 safety plan as the Rounds will be delivered in the context of the COVID-19 pandemic restrictions. It describes the delivery of the Rounds in the unit-based format that may provide additional benefits in the areas of team cohesion and sense of a shared purpose for participating units.

### Study Aims and Objectives

The primary study objective is to examine the feasibility of the Rounds in an Australian setting through the assessment of participant feedback provided via postround evaluation surveys and focus groups, Rounds’ attendance, and fidelity.

The secondary study objective is to evaluate the impact of Rounds on improving staff well-being, compassionate care, and organizational culture.

The research questions we explore within the study are as follows:

- What are the factors that underpin feasibility of Rounds?
- What impact does Rounds have on staff well-being, compassionate care, and organizational culture?

## Methods

### Study Design and Setting

This prospective pilot study will utilize a mixed methods, uncontrolled, repeated measures design. This design was selected to test the feasibility and preliminary efficacy of the Rounds and inform possible adaptations of their content and structure that may be needed in future larger studies. The study will be conducted in Mater Hospital, an Australian tertiary metropolitan hospital that offers a wide range of medical, surgical, and mental health services for adolescents and adults.

### Participant Selection and Recruitment

As this is a pilot study of Schwartz Rounds in hospital settings, main eligibility criteria for participation in the study will include being employed at Mater Hospital and willingness to participate in all aspects of the study (focus group participation is not mandatory). Members of the research team and planning committee will be excluded from participation in the study to avoid any risk of bias. Participants will include both clinical staff (ie, nurses, doctors, allied health) and nonclinical staff (ie, administrative/managerial, catering and ward services, health security, pastoral care) employed at Mater Hospital.

Staff interested in participating in the Rounds will receive an email from the department head that will include the research flyer and the participant information and consent form, and they will be asked to return the signed consent form to the research team via email. Potential participants will be given the opportunity to receive further information and ask questions to the research team regarding the study over the phone or via email. Once consent forms have been received, participants will be sent a link to the online baseline survey to complete prior to commencement of Rounds. The surveys will be distributed by the study research assistant, via email, 1-2 weeks prior to each Round and returned by the day of the Round. Facilitators will meet with the panel members 2-3 weeks prior to each Round, for 1-2 hours, to discuss the topic and familiarize them with the Rounds processes.

**Figure 1:**
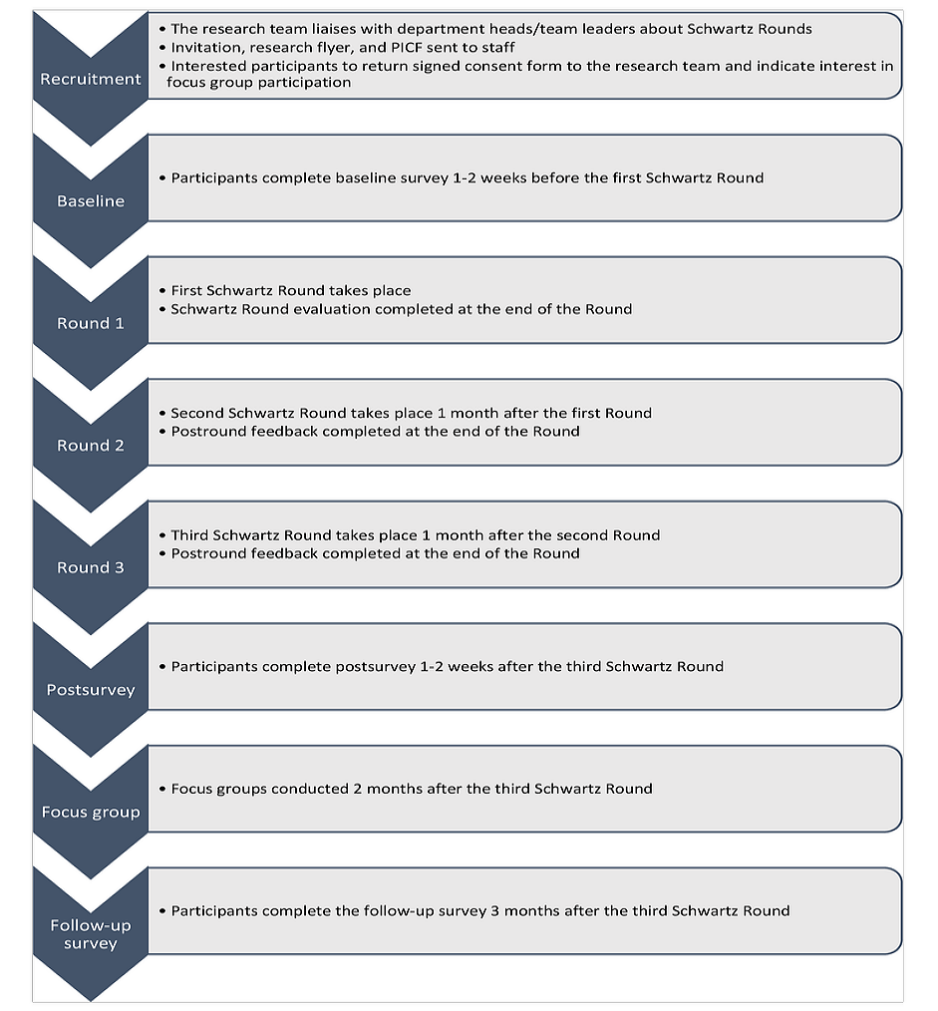
Flow of participants through the study. PICF: Patient Information Consent Form.

During the study planning, the research team liaised with several Mater Hospital departments, and based on the staff interest and teams’ needs, selected 2 teams to participate, the intensive care unit (ICU) and the gastroenterology department. The study participants will therefore be recruited in 2 groups, the ICU group and the gastroenterology group, who will complete 3 unit-based Rounds each. The participants will complete a quantitative survey focusing on staff well-being, compassionate care, and organizational culture at baseline, after the Round, and 3 months’ follow-up. They will also complete a short postround feedback form after each Round that incorporates both Likert scale questions and an open-ended comment section. During consenting, participants will be invited to participate in a focus group session to provide detailed feedback on the Rounds. The flow of participants in the study is shown in Figure 1.

### Schwartz Rounds Intervention and the Rounds Process

Each Round will follow the format outlined by the Schwartz Centre [10]. A steering committee will be made up of the research team members (facilitators, clinical leads, an administrator, and a research assistant/project manager), the members of Mater HR, and other Mater staff champions (ie, team leaders/department heads, executive committee). The research team will meet fortnightly to discuss the planning and delivery of the Rounds and other research-related activities. A steering committee will meet monthly to quarterly, depending on the need. Each member of the research team will be trained in how to run the Rounds in alignment with the standard procedure provided by the Schwartz Centre for Compassionate Health Care [10].

Facilitators will emphasize that the purpose of the Rounds is not to solve organizational problems or clinical management issues, but to provide a psychologically safe space where staff can speak freely about the social and emotional aspects of working within health care settings. Patient and staff confidentiality will be highlighted, and participants will be informed that they are to maintain confidentiality of the content of Rounds. To ensure the privacy and confidentiality of the staff, confidentiality will be discussed at the beginning and closing of each Round as well as in the panelists’ preparatory sessions and focus groups. Each Round will take approximately 1 hour. Facilitators will allow time for participants to ask questions and engage in open discussion, to provide opportunity to reflect on the stories shared, and to discuss their experiences of hearing the stories of their colleagues.

The Rounds will be held in-person during lunch or another time suitable for participating staff. Staff will also be invited to participate as Round panelists. A panel of speakers, usually 2-4 staff members from different work areas, will spend a combined 15 minutes presenting a patient story or talking about a topic related to their work. Panelists will have an opportunity to practice their story with the facilitators before the Round is conducted. They will be debriefed after each Round to help achieve closure and identify any immediate feedback or concerns.

Immediately after the completion of each Round, participants will be required to complete the Schwartz Centre Evaluation. After each Round, facilitators will complete a brief fidelity checklist and the research team will meet to discuss any immediate feedback. After the completion of 2 Rounds, an online survey link for the postrounds survey will be emailed to participants with a request to be completed within 1-2 weeks. Participants will be asked to complete surveys at baseline, after completing the Rounds, and at 3 months after the Rounds; attend at least two out of three Rounds; and provide immediate postround feedback. In addition, 3 participants per Round will be invited to participate as a panelist and share their stories and experiences in health care, making up the content of the Round.

To test the recruitment, delivery, and assessment procedures and inform any necessary adaptations prior to commencement of the study, the research team conducted an in-house pilot Round with the consultation liaison team. The 3 panelists, a psychologist, psychiatrist, and a psychiatric registrar, in discussion with the facilitators, agreed on the Round topic “When compassion is hard to find when caring for patients and their families.” The pilot Round was attended by 21 participants from a variety of nonclinical and clinical disciplines and their feedback on the Round as well as the study procedures was used to fine-tune the recruitment and data entry processes and incorporated in the study protocol. The participants’ feedback was overwhelmingly positive with 100% (21/21) of attendees reporting that the pilot Round gave them new insights into the perspectives and experiences of their co-workers, 86% (18/21) reporting new insights into the perspectives and experiences of their patients, 67% (14/21) reporting feeling better prepared to handle tough or sensitive situations, 76% (16/21) feeling less isolated in their work, and 95% (20/21) stating that they planned to attend future Rounds.

### Data Management and Analysis

Qualitative and quantitative data will be collected by the research team through the self-report questionnaires, Rounds attendance sheets, postround feedback forms, and focus groups transcripts. Data will be deidentified and each participant will be given a number or pseudonym. All deidentified and identifiable data will be stored electronically on REDCap and Griffith University Research Space, where only the research team will have access to the data. All hard copy documents will be stored in a locked cabinet at Griffith University where only those that require direct access will be able to access it. Electronic information will be kept on encrypted devices. All data will be stored for 5 years and then destroyed in compliance with Griffith University and Mater Health policies. Upon study completion, deidentified data will be kept on the Griffith University repository after the final ethics report has been submitted.

Qualitative data from the open comment section of the standard postround evaluation form and focus groups transcripts will be thematically analyzed by 2 members of the research team [26]. Quantitative data will be analyzed using SPSS (IBM). Descriptive statistics, paired *t* tests, chi-square tests (or other suitable nonparametric tests), and analysis of variance will be used to compare survey data across time points and groups. Post hoc analyses will be conducted to compare differences between specific time points.

### Outcome Measures

Participants will complete an online survey via REDCap at preround, postround, and 3-month follow-ups, which will collect demographic information and measures of staff well-being, compassionate care, and organizational factors. Demographic data will include age, sex, ethnicity, profession, length of time in profession, workload and typical roster, and length of employment at the Hospital.

Staff well-being will be measured using the Maslach Burnout Inventory–Human Services Survey (MBI-HHS) [27]. The MBI-HHS is a reliable and validated 22-item measure that measures burnout using 3 subscales: Emotional Exhaustion (α=.90), Depersonalization (α=.79), and Personal Accomplishment (α=.71), on a 7-point scale ranging from “never” to “everyday.” Compassionate care will be measured using the Schwartz Centre Compassionate Care Scale (SCCCS) – provider version [28,29]. The SCCCS consists of 12 items answered on a 10-point scale and has been shown to be reliable and valid for use with patients (α=.98) [29]. Organizational culture will be assessed using the Culture of Care Barometer – version 2 (CoCB-v2) [27]. The CoCB-v2 is a reliable and valid measure that consists of 30 items across 4 subscales: Organizational Values (α=.93), Team Support (α=.93), Relationships With Colleagues (α=.84), and Job Constraints (α=.70) [30,31].

In addition to these outcome measures, participants will be asked to complete the standard Schwartz Rounds Evaluation Survey at the end of each Round. This survey includes 10 questions answered with no, yes, or not sure; a question asking the participants to rate the Round overall as either poor, fair, neutral, good, or excellent; a free text question asking the participants to list 2 ways in which the Rounds will change how they related to or communicate with patients or colleagues; and an open comment section. Textbox 1 shows the Schwartz Rounds evaluation survey.

**Schwartz Rounds evaluation.**

- During this session, attendees discussed challenging social and emotional aspects of patient care.
- Today’s discussion gave me new insights into the perspectives and experiences of my co-workers.
- Today’s discussion gave me new insights into the perspectives and experiences of patients or families.
- As a result of this discussion, I feel better prepared to handle tough or sensitive patient situations.
- As a result of this discussion, I feel less isolated in my work with patients.
- As a result of this discussion, I feel more open to expressing thoughts, questions, and feelings about patient care with colleagues.
- I plan to attend Schwartz Center Rounds again.
- The discussion was well-facilitated.
- Today’s discussion suggests that changes may be needed in departmental or institutional policies or practices.
- Today’s program was free of commercial bias.
- Please rate today’s program overall.
- List 2 ways in which today’s discussion will change how you relate to or communicate with patients or colleagues (if any):
  1. (Example)
  2. (Example)
- Additional comments, including ideas for future topics: (If you would like to share input with the Planning Committee member about your ideas, please provide your name and contact info.)

Focus groups will be conducted after the completion of the Rounds, to give participants the opportunity to provide detailed feedback on the Rounds. Focus groups will be facilitated by an external member of the research team with prior experience in facilitating groups and adequate knowledge of the topic and the purpose of the group. Conversation openers developed by the research team will be used [14-16]. Focus groups will be conducted approximately 2 months after the third Round for each group. Qualitative data will be analyzed thematically by 2 researchers (GH and AT) independently to ensure validity.

### COVID-19 Risk Mitigation Plan

The impact of conducting Rounds in the context of COVID-19 challenges was discussed regularly during the study conception and planning. Research team considered the use of virtual Rounds in the planning stage but decided to proceed with the in-person Rounds, in view of the confidentiality and sensitivity and strong participants’ preference for in-person attendance that was indicated after the pilot Round.

Rounds planning included considerations of the reduced staff participation due to COVID-19–related quarantine, sick and family leave, low staffing levels on Hospital wards, and difficulties in accessing adequate room space to conduct the Rounds safely in accordance with COVID-19 social distancing requirements. The study COVID-19 risk mitigation plan specifies the personal safety and hygiene measures to be followed by the research team and participants, in compliance with Queensland Government and Mater Health COVID-19 guidelines. The measures include social distancing and may include mask wearing and rescheduling of the Rounds during acute lockdowns, in keeping with the government and organizational directives at the time the Rounds are scheduled.

### Safety Considerations

Possible risks to participants include a potential risk of unintended psychological harm to some participants, if they experience emotional distress in response to Rounds content. This risk will be minimized by having 2 researchers (GH and TE) facilitate the Round and 2 members of the research team (JM and AT) attend each Round to focus on participants and monitor for emotional distress. Contact information of the research team will be provided to discuss issues and, an external advisor, experienced in the running of Rounds in Australia, will be engaged in a consultative role. Prior to Rounds, all participants will be given brochures on the Hospital employee assistance program to access counseling and support if needed. In the event of harm occurring through damaged networks with peers or the organization, confidentiality will be emphasized.

### Sample Size and Statistical Power

Participants will be recruited via a convenience sample. The required sample size was calculated using a power calculation for a repeated measures analysis of variance (one group measured across three time points) study using G*Power with effect size of 0.25 (*f*), α level of .05, power of 0.80, and correlation among repeated measures of 0.5. Therefore, it is calculated that a total sample size of 24 will be required for the study to be adequately powered to detect a significant difference (*P*<.05) between the time points. Approximately 10-15 participants will be selected from interested staff, to participate in 2 focus groups. This number of participants will ensure that 5-8 participants will take part in each focus group, in keeping with the recommended size for focus groups, and to ensure an even spread between professions, consistent with previous research recommendations [15,22].

### Reflexivity to Improve Robustness

Prior to commencing the research, each member of the research team wrote and shared with the team a reflexive statement describing their prior experiences, assumptions, and beliefs about the research process and Schwartz Rounds. These statements will be discussed and revisited in regular research team meetings to enhance the individual researchers and team reflexivity, and the research integrity of the study [32].

In sharing their reflexivity statements, all members of the research team identified that they believed that the Rounds would enhance staff well-being and contribute to more sustained compassionate care and rejuvenated progressive organizational culture. Some members of the research team considered how their dual roles of being a health professional/clinical leader and a member of the research team might affect the research process. It was also acknowledged that, because of the hierarchical nature of the health care system, staff could feel pressured to participate in the Rounds when an operational or clinical manager was promoting the Rounds. Furthermore, concerns about the busy Hospital staff being released to attend the Rounds were identified. These concerns highlighted the importance of the research team members and steering committee communicating regularly with the Hospital executive team and directors of the departments involved in the study to promote the value of the Rounds. Finally, research team members acknowledged that changing organizational culture would take time and that this pilot project would ideally be a springboard for establishing ongoing Rounds in the Hospital.

### Patient and Public Involvement

Views on the study design were obtained during the study conception from the clinical staff working in different departments as well as the nonclinical staff including administrative staff, security workers, human resources staff, and pastoral care. Patient views on the potential benefits of Hospital staff participating in an intervention facilitating compassionate care and staff well-being were elicited through the Hospital Consumer Consultancy Group members. Although the study participants were not formally involved in the recruitment or data analysis, department leaders and other staff members were consulted regarding their departments’ participation.

### Ethics Approval

The study was approved by the Mater Hospital Human Research Ethics Committee (reference number: HREC/MML/71868).

## Results

This study was funded by the Griffith University Health Group collaboration grant in October 2020 and approved by the Mater Hospital Human Research Ethics Committee on January 13, 2021. Recruitment commenced in July 2021 and was completed in September 2021. Data collection commenced in July 2021, with projected completion by March 2022. Data analysis will commence in April 2022 and the results are expected to be published in the second half of 2021. The trail has been registered in the Australian New Zealand Clinical Trials Registry (ACTRN12621001473853).

## Discussion

The key goals of this study are to evaluate the feasibility and efficacy of the Rounds in improving compassionate care, staff well-being, and organizational culture in a tertiary, metropolitan Australian hospital where the cultural background might be different to that of the North American or United Kingdom settings. Although the Rounds are currently being conducted in several other Australian locations, this study is, to our knowledge, the first pilot research study of Schwartz Rounds in Australia.

Another important feature of the study is the Rounds delivery during the current COVID-19–related challenges where this type of staff wellness intervention could be crucial in mitigating staff burnout and improving patient care and organizational culture. Furthermore, the implementation of the Rounds in unit-based format may demonstrate the feasibility and benefits of the Rounds in the most acutely affected departments such as the ICU as well as those with secondary overflow and staffing issues such as the gastroenterology department. Unit-based Rounds may also be more acceptable to staff and more likely to enhance teamwork and sense of shared purpose than the hospital-wide Rounds due to their smaller size and more cohesive group composition.

The use of the mixed methods, combining qualitative data from 2 different sources with quantitative data from the MBI-HHS, SCCCS, and CoCB-v2, will enhance the methodological rigor of the study and the confidence in its findings. The study findings have the potential to provide novel insights into the factors underpinning the feasibility and efficacy of the Rounds and their mechanism of action, and may inform the refinements of the Rounds in future studies.

Key limitations of the study are related to the relatively small sample size and a lack of a control group, which may lead to statistically insignificant findings in some outcome measures and limit the study’s generalizability. However, given the pilot and exploratory nature of the study, its primary role is the assessment of the feasibility of the intervention and study procedures, with qualitative data providing additional valuable insights into these areas.

The results of the one-off pilot Round conducted in January 2021 provided a promising early indication of the potential value of Rounds in Australian settings, and during significant COVID-19–related challenges. The study is, to our knowledge, the first to evaluate Schwartz Rounds in a unit-based format during the COVID-19 pandemic and the first to include the departments potentially acutely affected by COVID-19. We anticipate that the results of the study will have a more wide impact as Schwartz Rounds are increasingly being adopted in different health care settings as well as in universities and other educational settings.

## Abbreviations

CoCB-v2: Culture of Care Barometer – version 2
ICU: intensive care unit
MBI-HHS: Maslach Burnout Inventory–Human Services Survey
SCCCS: Schwartz Centre Compassionate Care Scale
